# Atmospheric drying as the main driver of dramatic glacier wastage in the southern Indian Ocean

**DOI:** 10.1038/srep32396

**Published:** 2016-09-01

**Authors:** V. Favier, D. Verfaillie, E. Berthier, M. Menegoz, V. Jomelli, J. E. Kay, L. Ducret, Y. Malbéteau, D. Brunstein, H. Gallée, Y.-H. Park, V. Rinterknecht

**Affiliations:** 1 Université Grenoble Alpes, LGGE, F-38041 Grenoble, France; 2 CNRS, LGGE, F-38041 Grenoble, France; 3 LEGOS, Université de Toulouse, CNES, CNRS, IRD, UPS, F-31400 Toulouse CEDEX, France; 4 Barcelona Super Computing Center, 08034 Barcelona, Spain; 5 Université Paris 1 Panthéon-Sorbonne, CNRS Laboratoire de Géographie Physique, F-92195 Meudon, France; 6 Cooperative Institute for Research in Environmental Sciences University of Colorado at Boulder 216 UCB Boulder, CO 80309, USA; 7 LOCEAN/DMPA, Muséum National d’Histoire Naturelle, F-75005 Paris, France; 8 University of St Andrews, DEES, St Andrews KY16 9AL, UK

## Abstract

The ongoing retreat of glaciers at southern sub-polar latitudes is particularly rapid and widespread. Akin to northern sub-polar latitudes, this retreat is generally assumed to be linked to warming. However, no long-term and well-constrained glacier modeling has ever been performed to confirm this hypothesis. Here, we model the Cook Ice Cap mass balance on the Kerguelen Islands (Southern Indian Ocean, 49°S) since the 1850s. We show that glacier wastage during the 2000s in the Kerguelen was among the most dramatic on Earth. We attribute 77% of the increasingly negative mass balance since the 1960s to atmospheric drying associated with a poleward shift of the mid-latitude storm track. Because precipitation modeling is very challenging for the current generation of climate models over the study area, models incorrectly simulate the climate drivers behind the recent glacier wastage in the Kerguelen. This suggests that future glacier wastage projections should be considered cautiously where changes in atmospheric circulation are expected.

Remote sensing data in the southern mid-latitudes have documented the occurrence of rapid and extensive glacial retreat^1^,^2^,^3^,^4^,^5^. However, full glaciological modeling is rarely used to identify the drivers of the ice wastage in these regions. Instead, interpretations are largely qualitative, based on simple comparisons with regional climatic trends. Recent modelling studies of the Patagonian Icefields are one exception^6^,^7^, but the studies only address changes in the surface mass balance since the mid-1970s and climatic interpretations were complicated by the prevalence of calving glaciers. The lack of full glaciological modeling studies has led to the main conclusion that, akin to northern sub-polar latitudes^8^,^9^,^10^,^11^, recent ice wastage at southern mid-latitudes can mainly be attributed to warming^1^,^2^,^3^,^4^,^5^, whereas no consensus has been reached on the role of possible changes in precipitation^1^,^5^,^6^,^7^,^12^. Because long-term, continuous, and reliable field measurements on and close to glaciers and ice caps are particularly scarce in the southern hemisphere mid-latitudes, conclusions on climatic trends and impacts are particularly uncertain.

Here we advanced understanding of the climate drivers of glacial wastage at southern mid-latitudes by analyzing variations in the Cook Ice Cap (CIC, covering 410 km^2^ in 2001) multi-decadal mass balance in the Kerguelen Islands in the southern Indian Ocean (49°S, 69°E, Fig. 1a). This ice cap is mainly composed of land-terminating glaciers and variations in its mass are more directly linked with climate variations than other ice caps with calving termini at the same latitudes. The continuous long term observations made since 1951 at Kerguelen (Port-aux-Français (PAF) station), plus dated geomorphologic evidence since the Little Ice Age (LIA)^3^,^13^,^14^, and glaciological observations since the 1970s, comprise an exceptionally long glacio-meteorological dataset. We used this unique set of long-term climate and glaciological observations in the southern sub-polar latitudes, the extended historical reconstruction of sea surface temperatures (SST) from ERSST.v2^15^ available since 1854, and old^3^ and new remote sensing mass balance measurements to constrain a mass balance model run from 1850 to 2011 to analyze the climatic drivers of the post-1960s retreat.

## Results

### Glacier wastage acceleration and modeling

**Figure 1:**
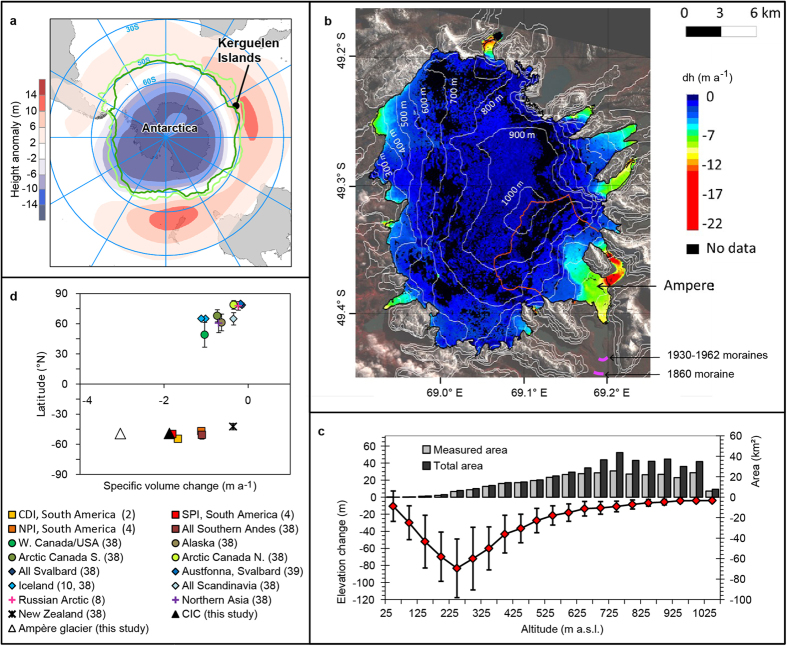
Climate settings of the Kerguelen Islands and mass balance of the Cook Ice Cap (CIC) between 2000 and 2009. (**a**) Map of the Kerguelen (black dot) and sub-Antarctic areas, with the mean positions of the sub-Antarctic (light green) and polar (dark green) oceanic fronts between 1993 and 2005 (ref. 26). The SAM 850 hPa geopotential height anomaly pattern is also shown as blue to red shading^70^. Map generated with ARCGIS 10.2 ArcInfo single use (http://www.esri.com/). (**b**) Rate of change in surface elevation (in meters per year) on the CIC. The extents of CIC and Ampère Glacier in 2009 are represented by the black and red lines respectively. Changes in elevation are measured with an accuracy of ±1 m (at the 1-sigma confidence level, see Methods). The locations of Ampère glacier moraines from 1930–1962 and from the 1860 as given in ref. 14 are also presented. Map generated with Quantum GIS 1.7.4-Wroclaw (http://www.qgis.org/). (**c**) Ice cap hypsometry in 2000 and changes in its elevation between 2000 and 2009 (with standard deviations for each 50-m elevation interval) as a function of elevation. (**d**) Latitude vs. changes in specific volume for different ice caps in the sub-Antarctic and sub-Arctic regions. The numbers associated with each ice cap correspond to the bibliographic references. Our results for CIC and Ampère Glacier are also plotted for the sake of comparison.

Geomorphologic evidence revealed that the front of the main CIC outlet, the Ampère Glacier (67 km^2^ in 2001), retreated 0.9 km between the LIA maximum in the early 1800 s and 1963. Between 1963 and 2003, the glacier retreated 2.8 km (i.e. 18% of its length), which corresponds to a 13-fold increase in its retreat rate since the 1960s (ref. 3). This accelerated retreat was accompanied by a major mass loss of the CIC^3^ (−1.33 ± 0.90 m water equivalent (w.e.) a^−1^) and a slight acceleration of ice loss during the 2000s (Table 1). The acceleration of ice wastage was revealed by subtracting two digital elevation models (DEM) computed from the Shuttle Radar Topography Mission (SRTM) in February 2000 and from a stereoscopic pair of SPOT5 images in December 2009 (see Methods, Fig. 1b), giving a total volume loss of 7.0 ± 0.4 km^3^. We computed an ice-cap wide mass balance of −1.59 ± 0.19 m w.e. a^−1^ between February 15, 2000 and February 15, 2010. The greatest ice loss occurred at Ampère Glacier, with a glacier-wide mass balance of −2.60 ± 0.30 m w.e. a^−1^. Thinning during the 2000 s was significant (0.4 ± 0.1 m a^−1^) right up to the summit of the ice cap (Fig. 1b,c).

**Figure 2:**
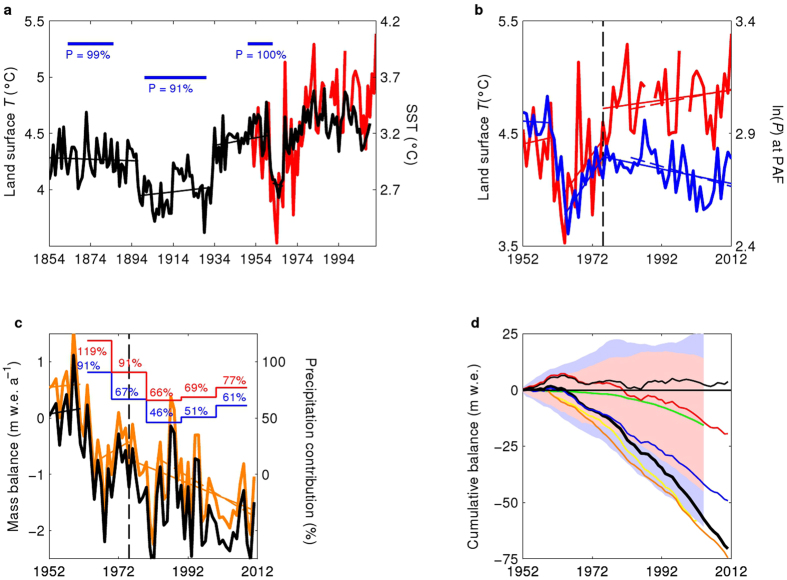
Climate drivers of the Cook Ice Cap mass loss. (**a,b**) Time series of historical surface ocean temperature from ERSST.v2 dataset (black line, **a**), of air temperature at PAF (red line, **a,b**) and the logarithm of precipitation at PAF (blue line, **b**). The horizontal blue lines in **a**) are periods of glacier front stability^14^ for which our glacier model was used to retrieve past precipitation amounts (as a % compared to the 1950s), to allow Ampère Glacier extent as given in Fig. 1b. (**c**) Time series of the modeled specific mass balance of CIC assuming that elevation and extent are those observed in 2009 (orange) and 1963 (black). Red (blue) lines and numbers are the contribution of precipitation to the negative glacier-wide mass balance for each decade, assuming that the conditions observed in the 1950s (respectively in the early 1900s) represent reference climatology. In the 1960s, the contribution of dryness reaches 119% because cool conditions reduced ablation (see Methods). The vertical dashed line represents the year 1975. The thin solid and dashed lines are trends computed over the timespan corresponding to the length of each line. (**d**) Time series of the cumulative modeled specific mass balance of CIC assuming that elevation and extent are those observed in 1963. The thick black line represents observations, the thin black line is hypothetical climate without warming or drying compared to the 1950s, the red line is without drying but with warming, the blue line is without warming but with drying. The yellow and orange lines are ERA40 and NCEP1 mass balances. The green line is the CMIP5 multi-model mass balance mean. The shaded areas are the spreading of all CMIP5 model values (blue area) and of 90% of CMIP5 model values (pink area). For each CMIP5 model, the temperature and precipitation biases with respect to observed data were removed based on the 1950–2005 period.

The recent acceleration of the retreat contrasts with the moderate fluctuations in the glacier fronts and temperature in the Kerguelen from 1850 to 1960 (Figs 1b and 2a). To identify the climatic drivers of the recent changes and to assess the relative importance of reduced precipitation and warming on CIC wastage, we forced a glaciological model with meteorological data from PAF (Fig. 2b) to compute distributed daily accumulation and ablation using a positive degree day (PDD) approach^16^. The daily surface mass balance model was combined with a simple ice motion routine^17^ to retrieve glacier snout positions (see Methods). Degree day factors were calibrated by comparison with calculated surface energy balance and continuous field measurements of ablation (see Methods, Supplementary Fig. 1). The modeled vertical mass balance profile is in good agreement with field measurements made between 1970–74 and in 2011 (Supplementary Fig. 2a). Modeled CIC mass balance values between 1963 and 2000 (−1.12 ± 0.39 m w.e. a^−1^) and between February 2000 and February 2010 (−1.62 ± 0.33 m w.e. a^−1^) are in remarkably good agreement with remotely-sensed estimates (Table 1). Considering the ice flow, model discrepancies are mainly concentrated on the western and north-western sides of CIC, where fewer data are available and glaciological processes are likely more complex. Given the complexity of modeling ice dynamics for small ice caps, the model satisfactorily reproduces the maximum CIC extent in the early 1960s (Supplementary Fig. 2b), demonstrating that it captures the main glaciological processes influencing CIC. Finally, the model reproduces acceleration in glacier wastage (see Table 1 and Fig. 2c): the 10-year mean mass balance was close to 0 in the 1950s (assuming extents and elevations from year 1963), very negative in the 1960s and 1970s (reaching −0.94 ± 0.39 m w.e. a^−1^), and increasingly negative up to the present.

### Climatic trends since 1850

Kerguelen is located along the seaway between South Africa and Australia, and historical ERSST.v2 data are based on a remarkably high number of SST measurements made since 1854 (Supplementary Fig. 3). The ERSST.v2 data show that warming was limited at Kerguelen (0.01 °C/decade) between 1850 and 1960, while average warming has been 0.13 °C/decade since the 1950s (Fig. 2a,b). Precipitation at PAF also displayed marked changes with a 50% reduction in annual precipitation between the 1950s and the 2000s (Fig. 2b), but precipitation amounts before 1950 are unknown. The glaciological model was driven to retrieve precipitation that would enable successive moraine deposits^14^ and a moderate shrinkage of the Ampère glacier between 1850 and 1960. Assuming that temperature anomalies during the 1850s were similar to those given for ocean surface by ERSST.v2, we forced the glaciological model with various precipitation anomalies (compared to the 1950s) until we reproduced the Ampère glacier front located 500 m downstream from the 1960s moraine^14^ (see Methods). The same approach was used with temperature anomalies in the early 1910s until we were able to reproduce the Ampère glacier front located 350 m downstream from the 1960s moraine. Modeling demonstrates that before the beginning of the CIC dramatic wastage in the 1960s, mean climatology ranged between the conditions observed in the 1950s (Fig. 2a) and the cooler dryer conditions observed around 1910, which may have produced the short term advance suggested by the small moraines deposited in the Ampère valley^13^,^14^.

The model was then driven using detrended temperature and precipitation time-series since the 1950s, showing that mean climatic conditions during the 1950s (i.e., without warming or drying) were consistent with glacier mass equilibrium (Fig. 2d). Thus, the decade 1950–1960 is a suitable reference period to analyze the contribution of precipitation and temperature to subsequent variations in the glacier mass balance.

### Glacier response to climatic trends since 1950

We then interpreted the chronology of glacier wastage^3^,^13^ after 1950. To quantify the drivers of glacier wastage following the equilibrium in the 1950s, the glacier model was forced with hypothetical meteorological datasets in which the observed warming and drying were successively removed after the 1950s (Table 1). We then compared the results of the glacier wastage computed with the real climate observed in each decade since 1960 (see Methods). Assuming ice cap hypsometry and extent is that in the year 2009, the model suggests that CIC would currently present a positive mass balance (0.58 mm w.e. a^−1^) if precipitation and temperature were at their 1950s level (Fig. 2c,d). Indeed, the shrinkage caused the loss of low elevation areas where ablation is the highest. In the 1960s, ablation was lower than in the 1950s and glacier wastage was entirely explained by dryness (Fig. 2c). Dryness was even more pronounced in the 2000s and despite the warming trend observed at Kerguelen since the 1960s, modeling suggests that 77% (between 75% and 81% for 1000 simulations with random degree day factors distributed according to a Gaussian law, see Methods) of the negative mass balance in the 2000s is still explained by dryness (see Methods, Table 1). This conclusion holds if we use the 1910s as a reference period to compute climate anomalies (Fig. 2c), demonstrating that the crucial role of precipitation variations in glacier-wide mass balance is independent of the choice of the reference period. The key role of precipitation was confirmed by the inconsistency between temperature and mass balance trend between 1963 and 1975, whereas the increase in precipitation is in perfect agreement with the increasing glacier mass balance at that time. The same conclusion can be deduced from trends after 1980. Thus, the decrease in precipitation was the first order driver of glacier mass loss, while atmospheric warming played a lesser amplifying role.

## Discussion

### Abrupt climate shift and accelerated glacier wastage

**Figure 3:**
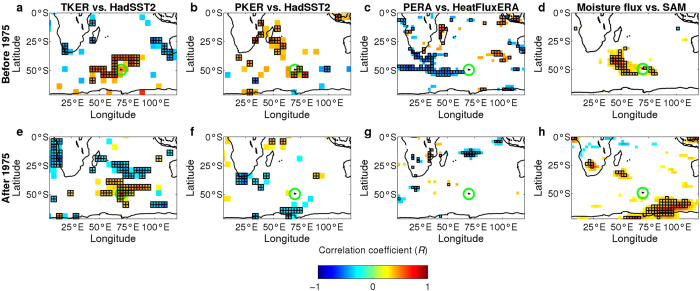
Teleconnections between the climate over the Kerguelen and conditions in the surrounding oceans. The correlation coefficient (*R*) was computed between December and March (DJFM) HadSST2 mean Sea Surface Temperature and DJFM mean local atmospheric temperature (**a,e**) and precipitation (**b,f**) observed at Kerguelen. (**c,g**) same as previous panels except for the correlation between ERA40 precipitation from the pixel including Kerguelen Islands and ERA40 surface latent heat flux during DJFM. Surface latent heat flux is negative when evaporation occurs. (**d,h**) Same as previous panels but between daily moisture anomalies and daily SAM anomalies during the winter accumulation season (from June to August). Anomalies were computed after removing the seasonal cycles and the data autocorrelation was removed to calculate the significance level. In (**d,h**) the correlations were multiplied by four to fit the color scale. Long term trends were removed from all the time series. Correlation maps are before 1975 (**a–d**) and after 1975 (**d–g**). Pixels for which the correlation is significant at 95% are in squared and crossed areas. Pixels for which the correlation is significant at less than 90% are not shown. The Kerguelen Islands are located inside the green circle. Maps were generated using Matlab R2011b (www.mathworks.com/products/matlab/).

An abrupt dry spell in the 1960s triggered glacier wastage. This dryness was related to large scale processes involving ocean-atmosphere interactions as suggested by the consistency between variations in surface air temperature and precipitation at PAF before 1975 (Fig. 2b), and between air and ocean surface temperatures from ERSST.v2 in the Kerguelen region (Fig. 2a). A correlation map between air temperature measured at PAF and SST data (HadSST2^18^) before 1975 shows a typical Subtropical Indian Ocean Dipole^19^ pattern (Fig. 3a), where positive phases are associated with high precipitation in South Africa due to the abnormally warm Agulhas current^19^. The SST of the Agulhas and Antarctic circumpolar currents along the southwestern limb of the south Indian subtropical gyre affect the evaporation rates within the storm track that eventually reaches Kerguelen (Fig. 3c), yielding positive correlations between precipitation on Kerguelen and SST along the Agulhas and Antarctic circumpolar currents (Fig. 3b). Before 1975, positive Southern Annular Mode (SAM)^20^,^21^ phases strengthened the westerly winds and moisture fluxes around the Kerguelen Islands (Fig. 3d). The inter-annual variability of precipitation (which is mainly orographic at Kerguelen) was thus significantly correlated with the SAM.

Changes in atmospheric circulation in the mid-1970s dramatically affected the link between temperature and precipitation in the Kerguelen. Reanalyses (ERA-40, NCEP-NCAR reanalysis1) show that drying and warming at Kerguelen since the 1970s are in pace with (i) a shift in the storm track (Fig. 3c,d,g,h and Supplementary Fig. 4). (ii) an increase in atmospheric surface pressure (Supplementary Figs 5a and 6), (iii) larger scale drying around Kerguelen and northward (Supplementary Fig. 7a,c), (iv) oceanic warming over large areas around Kerguelen (Supplementary Fig. 8). As a consequence, the post-1975 correlation map between HadSST2 data and temperature at Kerguelen displays a dipole-like pattern but with a south-north orientation (Fig. 3e) that differs from the pattern observed before 1975 (Fig. 3a). The correlation map for precipitation changed dramatically after 1975, showing a negative correlation between Kerguelen and regions to the south of South Africa (Fig. 3f), suggesting that intensity of precipitation on the Kerguelen is no longer affected by the Agulhas and the Antarctic circumpolar currents system (Fig. 3f). High pressure conditions shifted the path of depressions southward, and Kerguelen is no longer under the direct influence of the storm track (Fig. 3g,h and Supplementary Fig. 4) but is impacted by the dryer conditions typically observed northward. Thus, the SAM values and the moisture flux are now correlated in regions located southward. Radiosonde observations at PAF (WMO ID 61998, 1973–2011) confirm a major reduction in moisture flux throughout the troposphere (between 1000 mb and 500 mb) since the 1970s (Supplementary Figs 9 and 10). The decreasing moisture flux reduced precipitation despite the increase in wind speed at Kerguelen, because the orographic effect on precipitations was less powerful. Climate change disrupted synchronicity between changes in precipitation and temperature after 1975, and trends were subsequently reversed (Fig. 2b). The frequency and intensity of precipitation events also decreased. This situation accelerated glacier mass loss after 1975 (Table 1, Fig. 2c) as well as the reduction in ice area since the 1990s (see Fig. 2 in ref. 3).

### Link with the SAM positive phases

The observed dryness over the Kerguelen is in agreement with the increasing shift of the SAM^20^,^21^,^22^,^23^,^24^,^25^,^26^,^27^,^28^,^29^ to its most positive phase (SAM+) over the last millennium^27^, and presents strong similarities with the situation described in New Zealand^30^. The SAM^29^ positive (negative) phase results in strengthening (weakening) of mid-latitude westerly winds. Precipitation is expected to increase during SAM+, but only if Kerguelen is within the storm track. SAM+ conditions also involve a poleward shift of the westerly winds^31^. The direct consequence is that the regions with high precipitation are now located south of the Kerguelen Islands.

Before 1975, Kerguelen was under the direct influence of the storm track, and moisture flux over the Kerguelen was correlated with the SAM index. Significant correlations were observed at every season and in particular in fall and winter (MAM and JJA (Fig. 3d)) when accumulation occurs on glaciers and ice caps. Precipitation on Kerguelen was thus strongly dependent on the SAM-index (r = 0.78, p = 0.0006, between 1960 and 1975). In particular, the post-1963 dryness was consistent with the low SAM-index values, which were probably caused by the massive eruption of the Agung Volcano^29^,^32^. However, this correlation disappeared after 1975. The following increasing positive phase of the SAM yielded increasing pressure anomalies over the Kerguelen and over large areas north of the Kerguelen (Supplementary Fig. 5a). This led to an anticyclone circulation mode anomaly in the Kerguelen region (Supplementary Fig. 6) resulting in a southward shift of the main wind regimes (Supplementary Fig. 5c). After 1975, the region impacted by the increase in moisture flux associated with the positive phase of the SAM shifted southward, closer to Antarctica (Fig. 3h).

### Inconsistency of climate models in the Indian Ocean

The recent SAM+ has been attributed to hemispheric-scale stratospheric ozone depletion, and increases in greenhouse gas emissions^20^,^22^,^23^,^24^,^27^. Over the next century, greenhouse gas emissions are expected to increase the frequency of SAM+ despite the recovery of the hole in the ozone layer^20^,^28^. Glacier wastage in the 21^st^ century is generally forecast^33^,^34^,^35^,^36^ using climate models from the Coupled Model Intercomparison Project 5 (CMIP5)^37^ exercise. However, we show that the ability of CMIP5 models^37^ to correctly simulate the drivers behind the changes in CIC is not robust at Kerguelen because precipitation modeling over the study area is very challenging for global atmospheric circulation models. We used outputs from historical simulations from 49 different versions of different models from 1950 to 2005 (Supplementary Table 1). Each of the CMIP5 models and the multi-model mean (MMM) suggest significant warming, but do not reproduce the decrease in precipitation in the Kerguelen region (Supplementary Fig. 7e). The decrease in precipitation is simulated in an area located 200 km north of the Kerguelen. The geographic location of the limit between the decrease and the increase in precipitation varies among the models, but only four models show a drying of more than 1 mm a^−1^ over Kerguelen for the 1951–2005 period whereas observations reported a drying of 7.3 mm a^−1^ over the same period. The models seriously underestimate the poleward expansion of the subtropical dry zone^28^ and are unable to capture the spatial pattern of observed precipitation trends in the Indian Ocean. To assess the magnitude and impact of this discrepancy, we forced our glaciological model using the temperature and precipitation anomalies given by each CMIP5 model. The results show that 95% of the models (Fig. 2d) underestimate the CIC glacier wastage. The MMM glacier mass loss is 84% lower than observed loss during the period 1963–2000, and similar to the hypothetical situation without drying (Fig. 2d). We conclude that CMIP5 long-term experiments poorly simulate the contribution of precipitation to recent glacier retreat at Kerguelen, and should be used with great caution in other regions of the Earth where major increases or decreases in precipitation are incorrectly reproduced by CMIP5 models (See supplementary Fig. 7).

The CMIP5 models predict substantially warmer and wetter conditions over the Kerguelen by 2100, even if precipitation does not return to its pre-1960 values (Supplementary Fig. 11). Because CMIP5 models incorrectly reproduced precipitation changes over the Kerguelen for the 20^th^ Century, we did not take the CMIP5 model precipitations into account in our model, but rather assumed that precipitation will progressively increase and reach the amount observed in the 1950s around the end of the 21^st^ century. This assumption forecasts seriously more humid conditions at the end of the century than projected by CMIP5 models. However, it makes it possible to account for conditions that should limit glacier wastage by the end of the 21^st^ century so that our projected mass loss for the ice cap can be regarded as a conservative (minimum) estimate. Despite this assumption, dramatic CIC wastage is expected to continue because the present-day 0 °C isotherm is already very close to the CIC summit, and further warming could eliminate snowfall and accumulation (except in the heart of winter) with important albedo feedback on melting (Table 1).

### Implication for the study of future climate change impacts

The CIC mass balance during the 2000s was amongst the most negative on Earth^2^,^4^,^8^,^10^,^38^,^39^ (Fig. 1d). Except for maritime icefields in Alaska^11^ and the southernmost ice caps of the Canadian arctic^40^, similar negative mass balances have only occurred at similar latitudes, for instance in the Darwin Cordillera^2^ and South Patagonian Icefields^1^,^4^, (Fig. 1d). The Southern Hemisphere subpolar and mid-latitudes are regions where glaciers are currently losing mass the most rapidly on Earth. Here, we demonstrate that drying, not warming, has been the main driver of the exceptional ice cap wastage in the Kerguelen. The drying was seen to start in the 1960s and was mainly driven by changes in the atmospheric circulation resulting from the anthropogenically-induced ozone hole and from global warming (via a SAM + situation). Due to the hemispheric SAM^20^,^22^,^23^,^24^,^25^,^26^,^27^ signature and to the related consequences for climate, the observed dryness may have affected other regions, with potential impacts on other ice bodies under the same latitudes. However, similar long term observations are not readily available elsewhere in southern hemisphere mid-latitudes to confirm this assumption. The availability of consistent glaciological and meteorological field observations since 1850 was what made it possible to reach this conclusion at Kerguelen. Shorter term (i.e. post 1960s) glaciological analyses or modeling based on reanalyses or climate models are doomed to fail in retrieving the origin of glacier wastage.

Our analysis demonstrates that glacier wastage cannot be estimated without accounting for changes in atmospheric circulation^41^ that affect precipitation rates. This has been demonstrated in the case of past abrupt changes in other regions, such as in the European Alps^42^ or in the Central Andes of Argentina and Chile^43^ during the LIA. We suggest that future ice losses^5^,^16^ based on CMIP5 should be considered with great caution where atmospheric circulation changes are expected^41^, in particular in the southern mid-latitudes where the SAM^20^,^22^,^23^,^24^,^25^,^26^,^27^ is expected to continue to have a strong influence.

## Methods

### Remotely-sensed estimates of mass balance

We measured ice elevation on the Cook Ice Cap (CIC) by differentiating between two digital elevation models (DEMs) generated in February 2000 (SRTM)^44^ and in December 2009 (SPOT5 optical stereo imagery^45^). A detailed description of the processing steps can be found in ref. 46 and are only briefly summarized here.

First, the SRTM DEM was bi-linearly resampled to 40 m, to match the resolution of the SPOT5 DEM. The planimetric adjustment was achieved by minimizing the standard deviation of the differences in elevation between the two DEMs in stable areas^46^,^47^. To account for the difference in spatial resolution of the two DEMs, we applied a correction derived from the off-glacier relation between differences in elevation and the maximum terrain curvature^48^. Given that the SAR images used to generate the SRTM DEM were acquired in mid-February 2000, the heart of the melt season in the Kerguelen Islands, and given the likely occurrence of liquid water in the snow, firn, and ice, we assumed no penetration of the SRTM C-band signal^3^,^49^.

Pixels interpolated in at least one of the DEMs (40% of the ice-covered areas) were excluded as were pixels in which the difference in elevation exceeded ± 150 m. Changes in glacier volume over void-filled regions of the DEMs were estimated assuming that void-filled pixels underwent the same mean change in elevation as measurable pixels in the same altitude interval. This value was added to the measured changes to obtain a total volume change in each altitude interval. Mean volume changes were then converted into glacier-wide mass balance by assuming a density of 850 kg m^−3^ (ref. 50). Because of the slightly different acquisition dates of the DEMs, we computed the balance from December 15, 2009 to February 15, 2010 with the PDD model (see below) and summed it to the volumetric mass balance. This resulted in a 0.08 m w.e. a^−1^ more negative mean mass balance.

Errors in the changes in elevation were estimated on ice free terrain where, by construction, the average elevation difference between the SRTM and SPOT5 DEMs is null (standard deviation = 4.6 m). A novel approach was developed here so that our error estimate captures the spatially-varying vertical biases in the DEMs^51^,^52^. The ice-free region surrounding the CIC was split into 25 tiles (5 × 5, we found little sensitivity of the error estimate to the number of tiles). Within each tile, N measurements of elevation changes were randomly drawn and averaged, leading to the average elevation difference μ_dh(i)_. N was chosen to “simulate” hypothetical glaciated areas of 410 km^2^ (the size of the entire CIC in 2000, N = 256250) and, respectively, 67 km^2^ (the size of Ampère Glacier, N = 41875). μ_dh(i)_ represents the error in the elevation changes for an ice body the size of the CIC (respectively Ampère Glacier) located within tile i. Then, the absolute value |μ_dh(i)_| was computed in each tile. Finally, the mean of the 25 values (one for each tile) of |μ_dh(i)_| was calculated and considered as our error in the difference in elevation. This error is ± 0.7 m for the entire CIC and ± 1.0 m for the Ampère Glacier. These errors are likely conservative, given that the mean glacier slope (12.5°) is more than twice steeper than the average slope on the CIC (5.3°) and errors in DEMs are known to increase almost linearly with slope^53^. During the conversion from volume to mass, we assumed an additional ± 60 kg m^−3^ error in the density conversion factor^50^.

### PDD modeling

Glaciological modeling was used to simulate the mass balance of the ice cap. Running a full surface energy balance model was not possible since the 1950s, because (1) long term radiation and wind speed measurements are only available at Kerguelen since the 1990s, (2) the reanalyzed (ERA40 and NCEP1) radiation and cloudiness data at Kerguelen did not significantly correlate with measurements made at Kerguelen since 1990 (data not shown).

Conversely, daily temperature and precipitation amounts have been measured since 1951 strictly respecting WMO protocols (Météo-France weather station) offering unique consistent long-term observations. A simple PDD model^54^,^55^,^56^ was used that allows calculation of daily snow or ice melt at a given elevation z, assuming two different degree day factor F for snow covered or bare ice. Temperature at distinct elevations is computed assuming a constant lapse rate (LR) with elevation but varying according to the orientation of the glacier. Modeled ablation was considered similar to melting, because sublimation is expected to be low at Kerguelen due to the high humidity^13^. Snow cover is the difference between ablation and snow accumulation at a given elevation *z*. Solid precipitation is assumed if the air temperature is below a threshold (Tsnow/rain = 1.0 °C (ref. 57)), otherwise solid precipitation is zero. A sensitivity test showed that higher values of Tsnow/rain do not affect the glacier wastage chronology, but a Tsnow/rain value of 2.0 °C causes the ELA to fall about 27 m and suggests a 0.27 mm w.e. a^−1^ less negative glacier-wide mass balance between 2000 and 2009 (assuming extents and elevations from the year 2009). A threshold of 1 °C led to a better agreement with remotely sensed mass balance estimates (Table 1) and with ELA estimates from MODIS images^56^. Mass balance modeling with the regional circulation model MAR (Modèle Atmosphérique Régional)^58^ for 2011 also suggests that Tsnow/rain = 1.0 °C is suitable for the PDD at Kerguelen. A spin up was applied to the PDD model, assuming that the climatic conditions observed during the first decade of the run were the same as the conditions that prevailed during the preceding decade.

### Calibration of degree day factors

The degree day factors were calibrated with ablation measurements made using stakes and with a sonic gauge. Modeled ablation was also compared to ablation estimated from surface energy balance computations, performed with data from two automatic weather stations (AWS) installed on the glacier (only available in summer) and close to the glacier, at La Mortadelle (Supplementary Table 2). Here, we used data from December 21, 2010 to January 4, 2011, and from December 14, 2011 to December 30, 2011. During these two periods, one of the AWS was located on the glacier, and measured albedo, air temperature and humidity and wind speed, allowing a direct comparison between daily ablation from the PDD model and from the surface energy balance. The model is fully described in ref. 59.

Modeling the surface energy balance makes it possible to reproduce measured ablation (Supplementary Fig. 1). The ablation values computed with the PDD and the SEB approaches were compared to calibrate the degree day factors F for snow and ice. Calibration of F suggests that:


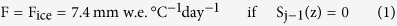


The calibrated F_ice_ is very close to the mean value at a global scale from ref. 16, i.e. F_ice_ = 7.2 mm w.e. °C^−1^ day^−1^. For snow, the calibration was performed on only five days with snow cover, leading to a value close to 5 mm w.e. °C^−1^ day^−1^. Due to the small number of ablation data used for this calibration, limited accuracy of this parameter was expected. However, the value is in good agreement with the mean F_snow_ = 4.9 mm w.e. °C^−1^ day^−1^given by ref. 16 at a global scale. Hence, we used the mean value from ref. 16 for situations with snow cover (see Supplementary Fig. 1):


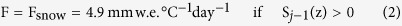


Model error resulting from uncertainties in the degree day factors was assessed from 1000 simulations with random degree day factors F for snow and ice distributed according to a Gaussian distribution centered on calibrated values and with standard deviations that equal 10% of the degree day factors. This represents approximately half the standard deviation of the degree day factors given at a global scale by 16, and leads to variations in model mass balance, with F values ranging from 5.0 mm w.e. °C^−1^ day^−1^ to 9.5 mm w.e. °C^−1^ day^−1^ for ice and from 3.4 mm w.e. °C^−1^ day^−1^to 6.2 mm w.e. °C^−1^ day^−1^ for snow. For each run, degree day factors were keptconstant with time.

### Temperature lapse rate and changes in precipitation with elevation

Field data and results from the regional circulation model MAR were used to get the regional distribution of temperature and precipitation. MAR presents the atmospheric scheme described in (ref. 58), coupled to a physically-based model of the snow pack^60^,^61^. The simulation was run at 10 km resolution on a stereographic grid for the year 2011. The model was forced with ERA-Interim reanalysis data, which is the most recent ECMWF reanalysis^62^, covering the period 1979 to the present. The surface elevation of each 10 km x10 km cell was assessed using the ETOPO1 global relief model^63^ to get accurate representations of the orographic impact on precipitation. Precipitation, temperature and elevation from each 10 × 10 km^2^ cell were extracted and compared with cell elevation. Precipitation amounts at different elevations and temperature vertical lapse rate (LR) from the MAR model were computed (Supplementary Fig. 12).

Eight different lapse rates were computed with the MAR model for eight sectors corresponding to the glacier azimuth (every 45°). For each azimuth, the LR value was assumed to be constant with elevation. The LR was calculated using the difference in temperature between the summit and the first pixel (in each of the eight directions) with more than 500 m difference in elevation. Results were compared to field data. Results from the MAR model gave a lapse rate of −8.3 °C km^−1^ along the glacier Ampère’s azimuth (South-East), of −8.8 °C km^−1^ eastward, while a LR of about −7.5 °C km^−1^ was modeled upwind of the ice cap. These values were checked with LRs obtained from the meteorological station located on the margin of the ice cap, on the glacier and at PAF, leading to a mean LR of −9.1 °C km^−1^ along Ampère Glacier, and of −8.6 °C km^−1^ for glaciers oriented to the East. Considering the good agreement between observation and the MAR data, we used the mean LRs given by the MAR for the eight sectors in our mass balance model.

We analyzed the impact of seasonal variations in the LR with the MAR. We observed that monthly variations in LR reach ± 1 °C km^−1^ (i.e. ± 15%). A stronger LR is observed leeward in summer, but this is compensated for by weaker LR in winter. Seasonal variations in the LR are weaker on the western side of the ice cap. We computed the CIC mass balance assuming the seasonal cycle given by the MAR, and obtained a 2.4% more negative mass balance for the period 2000–2010, which lies well within the PDD modelling error bars associated with differences in the surface and elevation in the first decade of the 21^st^ century.

We also used the MAR model to assess the spatial pattern of precipitation. We found a significant correlation between precipitation and elevation (Supplementary Fig. 12). The relationship, extrapolated to sea level, agrees with precipitation data at PAF (Supplementary Fig. 12b) even if this relationship underestimates the precipitation amounts at La Mortadelle, because the latter site is located in a small corridor and appears to present site specific precipitation amounts. The relationship between elevation and precipitation was used to force the PDD model at different elevations.

### Distributed modeling with cellular automaton

A cellular automaton^64^ was coupled to the PDD modeling to reproduce ice motion from high elevations down to the glacier snout. This method is widely used in many paleo-climatic studies^17^,^65^. The model considers that ice is a plastic material moving through avalanches and deformation/sliding. Glacier motion was reproduced assuming that the basal shear stress was maintained at a constant value of one bar^66^. The cellular automaton operates discrete ice movement on a hexagonal grid, allowing six possible cardinal directions of movement. The flow is thus largely controlled by the local slope. If the depth-slope product of the accumulated ice exceeds a threshold basal shear stress, deformation/sliding is simulated by moving sufficient ice to the lowest adjacent nodes to reduce the slope and depth. The ice flux field is determined by mass conservation and ice velocities are derived from this flux and are therefore closely related to the idea of balance velocities. The model poorly reproduces ice velocities, and is not appropriate to analyze glacier response times or transient glacier regimes. The simulations were performed over a period of 200 years, ensuring that the glacier reached equilibrium with the climatic conditions. Finally, the model does not account for calving processes. However, in 2009, the ice cap only counted one tidewater glacier and six lake terminating glaciers out of a total of 17 glaciers. Examination of satellite images showed that icebergs are rare close to the glacier fronts, suggesting that calving processes are not a first order cause of the ice cap wastage, even though it may have impacted the behavior of particular glaciers including Ampère Glacier.

The ice flow model was tested to check that the computed glacier-wide mass balance in the early 1960s was in agreement with the CIC stability. The ice flow model reproduced the maximum CIC extent in the early 1960s (refs 3,14) (Supplementary Fig. 2b), demonstrating that the glaciological model satisfactorily reproduces the main glaciological processes on CIC.

### Precipitation and temperature estimates since 1850

We propose that the 1950s can be used as a reference period because the modeled mass balance during this period was close to zero and because the glacier wastage began in 1963, suggesting that main climate changes occurred after this date. This is in agreement with the stability of the Ampère Glacier front observed in the 1950s (Fig. 1b), and with the limited retreat observed since the LIA^13^,^14^. However, ten years is a relatively short reference period. To justify this choice, we examined the observed change in temperature and the inferred change in precipitation in previous decades.

Air temperature fluctuations before 1950 were examined using the ERSST.v2 historical ocean surface data because they offer an extended image of possible past changes in ocean surface temperature. The ERSST.v2 data are based on the Comprehensive Ocean-Atmosphere Data Set (COADS) dataset, which consists in quality controlled marine surface observations from ships, moored environmental buoys, drifting buoys, and near-surface measurements from oceanographic profiles^15^. The ERSST.v2 dataset is generated using *in situ* SST data (falling within 2° latitude × 2° longitude boxes) and improved statistical methods that allow stable reconstruction despite sparse data, and provides monthly statistics of basic marine variables for each year. Because ERSST.v2 relies on only sparse historical data, we examined if ERSST.v2 data in the Kerguelen area were based on a significant number of *in situ* measurements before the 1970s (Supplementary Fig. 3). We checked that this SST dataset is representative of the temperature in the Kerguelen Islands, as suggested by the significant correlation between annual values (r = 0.76, n = 58, p < 10^−8^). A significant correlation (r = 0.97, n = 227, p < 10^−8^) between monthly ocean surface temperatures from a buoy at PAF, in the Gulf of Morbihan (ROSAME network) and monthly mean 2-m air temperature at PAF between 1993 and 2013, confirms that air and ocean surface temperatures are very closely related in this area. The ERSST.v2 (Fig. 2a) data confirm that the temperature has been relatively stable since 1935, whereas the cold situation following 1963 appears as short term variability, probably caused by the eruption of the Agung Volcano^29^,^32^.

We reconstructed precipitation data until 1854. We forced the glaciological model with temperature anomalies obtained between ERSST.v2 data from 1935–1962 and from other selected periods (Fig. 2a), and searched for the precipitation amounts that would enable the building of moraines observed during previous steady state periods of the glacier since 1854. Assuming the 1854–1890 temperature anomaly, we observed that the building of the moraine in the 1860s (ref. 14) was possible if precipitation was only 1% less than in the 1950s. Slightly dryer conditions (9%) were observed in the periods 1892–1913 and 1925–1934 (the coldest period in ERSST.v2 data) when cooler situations occurred in pace with glacier advance, as suggested in ref. 14.

### Contribution of precipitation and temperature changes to variations in mass balance

The contribution of changes in precipitation to variations in mass balance (as reported in Fig. 2c) was quantified for each decade from these simulations using the following percentage:


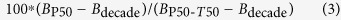


where *B*_decade_ is the mass balance calculated using precipitation and temperatures observed during the decade of interest, *B*_P50_ is the mass balance calculated with precipitation from the 1950s, i.e. without drying, and *B*_P50-T50_ is the mass balance calculated with precipitation and temperatures from the 1950s without either drying or warming. If we take the example of the 2000s (Table 1) when *B*_decade_ = −1.62 m w.e. a^−1^; *B*_P50_ = 0.07 m w.e. a^−1^; *B*_P50-T50_ = 0.58 m w.e. a^−1^, the above ratio is 77%. The contribution of changes in precipitation may exceed 100% (like during the 1960s, Fig. 2c) if low temperatures reduce ablation. This calculation was repeated for 1000 simulations with random degree day factors as described above. The contribution of precipitation changes to the mass balance in the 2000s ranged between 75% and 81%.

We checked that the use of the 1950s as a reference period did not impact our conclusion concerning the role of precipitation. Indeed, abnormally wet conditions in the 1950s would lead to artificial overestimation of the role played by decreasing precipitation in glacier wastage. Since slightly colder and dryer conditions were observed in the early 1900s, Equation (3) was also applied using hypothetical meteorological conditions in the periods 1892–1913 and 1925–1934 (hereafter referred to as climatic conditions in the early 1900s) as a reference, instead of the conditions observed in the 1950s. Our conclusion that precipitation played a key role in glacier wastage since 1960 remains unaltered if these different reference periods are used (see results of climatic conditions in the early 1900s in Fig. 2c).

### Reanalysis and climate models data used in this study

In addition to temperature and precipitation data from the meteorological station at PAF (Fig. 2), the ERA-40 from the ECMWF and NCEP-NCAR Reanalysis1 (NCEP1) were analyzed to confirm that drying is a large scale phenomenon around Kerguelen. ERA-40 fully covers the period 1958–2001^67^ with a nominal resolution of 125 km. NCEP-NCAR Reanalysis1 (NCEP1) are global atmospheric reanalyses available from 1948 to the present^68^ with a spectral resolution of 210 km. The outputs from the Coupled Model Intercomparison Project 5 (CMIP5)^37^ were also analyzed here to test whether these models are able to reproduce drying over the Kerguelen, and to check if model outputs, when used to force the glaciological model, are able to reproduce the trend of recent glacier wastage in the Kerguelen. CMIP5 long-term (or historical) simulations on a century time-scale involve 20 climatic groups using atmosphere-ocean global climate models (AOGCMs, sometimes coupled to a carbon cycle model) initialized from the end of freely evolving simulations of the historical period^37^. Outputs from historical simulations, and a multi-model mean (MMM) were computed from 49 different versions of different models (interpolated on ERA-40 grid) (Supplementary Fig. 7, Supplementary Table 1).

Projected precipitation and surface temperature linear trends from 2006 to 2100 for the CMIP5 MMM and for models showing the highest drying and warming trends over the Kerguelen under the lowest (RCP 2.6) and the highest (RCP 8.5) scenarios are presented in Supplementary Fig. 11. CMIP5 MMM clearly suggests significant warming by the end of the 21^st^ century (+0.0073 ± 0.0115 °C a^−1^ in the RCP2.6 scenario and + 0.0296 ± 0.0200 °C a^−1^ in the RCP8.5 scenario) and only a slightly increasing trend in precipitation (+0.51 ± 1.35 mm a^−1^ for RCP2.6 scenario and +1.92 ± 1.86 mm a^−1^ for RCP8.5 scenario) over the Kerguelen.

### Modeling based on CMIP5 model outputs

We tested the ability of ERA-40, NCEP1 reanalyses and of the CMIP5 exercise to correctly simulate climate over the Kerguelen. To test the impact of climate model discrepancies on glacier modeling, precipitation and temperature from each CMIP5 model and from ERA40^67^ and NCEP1^68^ reanalysis were used to force the glaciological model. Here, we used outputs for the period 1951–2005. Modeled temperature and precipitation means and trends from each model and reanalysis were compared to observations to deduce anomalies. Because model outputs are largely biased in Kerguelen region, the temperature bias over the period 1951–2005 was removed from each model by subtracting the mean bias, whereas precipitation rates were divided by the mean ratio between observations and the model. Using this procedure, the mean forcing values were correctly reproduced, and the mean glacier wastage was correctly modeled, but the mass loss acceleration was only correctly modeled if precipitation and temperature trends were close to observations. This was confirmed by the good agreement between modeled mass balance based on ERA40^67^, NCEP1^68^ corrected trends and the observed mass balance (Fig. 2d). Only three models (i.e. FGOALS-G2, MRI-ESM1, and MRI-CGCM3) give similar cumulative mass balances to observations. However, FGOALS-G2 suggests increasing precipitation. MRI-ESM1 and MRI-CGCM3 suggest seven times lower drying trends than observations. Moreover, these models give more negative glacier mass balances in the 1960s but less negative in the 2000s than observations. As a consequence, they fail to reproduce the accelerating trend of glacier mass losses. An in depth analysis of precipitation events in models showed that (1) the distributions of precipitation intensity in the models differ significantly from observations, and, (2) even the MRI-ESM1 and MRI-CGCM3 models, which are considered the most comprehensive models in the Kerguelen region, do not reproduce the reduction in precipitation frequency and intensity observed in field data^56^. Finally, a distinct careful model selection was performed according to ref. 69 to retrieve the models that best reproduce the mean temperature, wind speed and moisture spatial distributions given by ERA-Interim at 700 hPa between latitudes 40°S and 60°S. This selection suggests that ACCESS1-3 is the best model between latitudes 40°S and 60°S. Yet, ACCESS1-3 largely underestimates warming (0.06 °C/decade) and drying (only 0.3 mm a^−1^) trends at Kerguelen leading to a largely underestimated glacier mass loss (−7.5 m w.e. over the period 1950–2005 compared to an observed value of −57.3 m w.e.).

## Additional Information

**How to cite this article**: Favier, V. *et al*. Atmospheric drying as the main driver of dramatic glacier wastage in the southern Indian Ocean. *Sci. Rep*. **6**, 32396; doi: 10.1038/srep32396 (2016).

## Supplementary Material

Supplementary Information

## Figures and Tables

**Table 1:** Measured and modeled mass balances for the Cook Ice Cap for different periods since the 1960s and at the end of the 21^st^ century.

Time period	Past and present climate			Future climate
	1958–63	1963–2000	2000–10	2090–99
Measured MB		−1.33 ± 0.90	−1.59 ± 0.19^i^	
Modelled MB (m we a^−1^)	−0.01 ± 0.24^ii^	−1.12 ± 0.39iii	−1.62 ± 0.33^iv^	
Modelled MB without warming^v^			−1.15 ± 0.33^iv^	
Modelled MB without drying^vi^			0.07 ± 0.33^iv^	
Modelled MB without drying nor warming			0.58 ± 0.33^iv^	
Mean multi-model MB (CMIP5)	−0.05 ± 0.24^ii^	−0.18 ± 0.39iii		−0.87vii/−10.6viii

^i^Volumetric mass balance from February 15, 2000, to February 15, 2010. Mass balance from December 15, 2009, to February 15, 2010 was computed with the PDD model and summed to the volumetric mass balance from remote sensing data to obtain the CIC mass balance for exactly ten mass balance years.

^ii^Uncertainty is the standard deviation of mass balance values of 1000 simulations assuming Gaussian distribution around the optimized degree day factors (see Methods).

^iii^Mean glacier-wide specific mass balance is the average of values obtained with extents and elevations from years 1963 and 2000 respectively. The uncertainty is the half-difference between minimum and maximum values summed to the model uncertainty described in ^ii^. The modelled mass balance is from February 15, 1963 to February 15, 2000.

^iv^Same as ^iii^ but with extents and elevations from years 2000 and 2009 respectively. The modelled mass balance is from February 15, 2000, to February 15, 2010.

^v^Temperature in the 1950s is reported to every following decade, whereas observed precipitation is used.

^vi^Precipitation in the 1950s is reported to every following decade, whereas observed temperature is used.

^vii^In this experiment, precipitation from the 2000s progressively increases and reaches values from the 1950s in 2100, i.e. twice the precipitation amount compared to today. This assumes a higher increase in precipitation than that suggested by CMIP5 models. Warming is given by the mean trend in CMIP5 models for the RCP2.6 scenario. This represents the expected least negative mass balance situation. Mean glacier-wide specific mass balance assumes surface area and elevation from 2009.

^viii^Same as ^vii^ but warming is from RCP8.5 scenario.
