# Supplementary material for: Atmospheric drying as the main driver of dramatic glacier wastage in the southern Indian Ocean
Source: Sci Rep. 2016 Sep 1;6:32396. doi: 10.1038/srep32396 (PMC5007672; doi:10.1038/srep32396)
Supplement: Supplementary Information [file srep32396-s1.pdf]

# Atmospheric drying as the main driver of dramatic glacier wastage in the southern Indian Ocean

V. Favier[(1,2)], D. Verfaillie[(1,2)], E. Berthier[(3)], M. Menegoz[(1,2,4)], V. Jomelli [(5)], J. E. Kay[(6)], L. Ducret[(3)], Y. Malbéteau[(3)], D. Brunstein[(5)], H. Gallée [(1,2)], Y.-H. Park[(7)], V. Rinterknecht[(5,8)]

[(1)] Université Grenoble Alpes, LGGE, F-38041 Grenoble, France
[(2)] CNRS, LGGE, F-38041 Grenoble, France
[(3)] LEGOS, Université de Toulouse, CNES, CNRS, IRD, UPS, F-31400 Toulouse CEDEX, France
[(4)] Institut Català de Ciències del Clima (IC3), 08005 Barcelona, Spain
[(5)] Université Paris 1 Panthéon-Sorbonne, CNRS Laboratoire de Géographie Physique, F-92195 Meudon, France
[(6)] Cooperative Institute for Research in Environmental Sciences University of Colorado at Boulder 216 UCB Boulder, CO 80309, USA
[(7)] LOCEAN/DMPA, Muséum National d'Histoire Naturelle, F-75005 Paris, France
[(8)] University of St Andrews, DEES, St Andrews KY16 9AL, UK

Correspondence to: V. Favier (favier@lgge.obs.ujfgrenoble.fr)

# Supplementary Information

## Supplementary Tables

**Supplementary Table 1. CMIP5 models and simulations used in this study.** Outputs are from the historical (Hist), and forecasted RCP2.6 and RCP8.5 experiments used in this study.

| *Model* | *Modelling group* | *Country* | *Hist* | *RCP2.6* | *RCP8.5* |
|---|---|---|---|---|---|
| ACCESS1-0 | Commonwealth Scientific and Industrial Research Organisation, and Bureau of Meteorology | Australia | y | n | y |
| ACCESS1-3 | Commonwealth Scientific and Industrial Research Organisation, and Bureau of Meteorology | Australia | y | n | y |
| bcc-csm1-1 | Beijing Climate Center, China Meteorological Administration | China | y | y | y |
| bcc-csm1-1-m | Beijing Climate Center, China Meteorological Administration | China | y | y | y |
| BNU-ESM | College of Global Change and Earth System Science, Beijing Normal University | China | y | y | y |
| CanCM4 | Canadian Centre for Climate Modelling and Analysis | Canada | y | n | n |
| CanESM2 | Canadian Centre for Climate Modelling and Analysis | Canada | y | y | y |
| CCSM4 | National Center for Atmospheric Research | United | y | y | y |
| CESM1-BGC | National Science Foundation, Department of Energy, National Center for Atmospheric Research | United States | y | n | y |
| CESM1-CAM5 | National Science Foundation, Department of Energy, National Center for Atmospheric Research | United States | y | y | y |
| CESM1-CAM5-1-FV2 | National Science Foundation, Department of Energy, National Center for Atmospheric Research | United States | y | n | n |
| CESM1- FASTCHEM | National Science Foundation, Department of Energy, National Center for Atmospheric Research | United States | y | n | n |
| CESM1-WACCM | National Science Foundation, Department of Energy, National Center for Atmospheric Research | United States | y | n | n |
| CMCC-CESM | Centro Euro-Mediterraneo per I Cambiamenti Climatici | Italy | y | n | y |
| CMCC-CM | Centro Euro-Mediterraneo per I Cambiamenti Climatici | Italy | y | n | y |
| CMCC-CMS | Centro Euro-Mediterraneo per I Cambiamenti Climatici | Italy | y | n | y |
| CNRM-CM5 | Centre National de Recherches Meteorologiques, Centre Européen de Recherche et Formation Avancée en Calcul Scientifique | France | y | y | y |
| CSIRO-Mk3-6-0 | Commonwealth Scientific and Industrial Research Organisation, and Queensland Climate Change Centre of Excellence | Australia | y | y | y |
| EC-EARTH | EC-EARTH consortium | Europe | y | n | y |
| FGOALS-g2 | Institute of Atmospheric Physics, Chinese Academy of Sciences, and Tsinghua University | China | y | y | y |
| FGOALS-s2 | Institute of Atmospheric Physics, Chinese Academy of Sciences | China | y | n | n |
| FIO-ESM | The First Institute of Oceanography, SOA | China | y | y | y |
| GFDL-CM2.1 | National Oceanic and Atmospheric Administration Geophysical Fluid Dynamics Laboratory | United States | y | n | n |
| GFDL-CM3 | National Oceanic and Atmospheric Administration Geophysical Fluid Dynamics Laboratory | United States | y | y | y |
| GFDL-ESM2G | National Oceanic and Atmospheric Administration Geophysical Fluid Dynamics Laboratory | United States | y | y | y |
| GFDL-ESM2M | National Oceanic and Atmospheric Administration Geophysical Fluid Dynamics Laboratory | United States | y | y | y |
| GISS-E2-H | National Aeronautics and Space Administration Goddard Institute for Space Studies | United States | y | y | y |
| GISS-E2-H-CC | National Aeronautics and Space Administration Goddard Institute for Space Studies | United States | y | n | n |
| GISS-E2-R | National Aeronautics and Space Administration Goddard Institute for Space Studies | United States | y | y | y |
| GISS-E2-R-CC | National Aeronautics and Space Administration Goddard Institute for Space Studies | United States | y | n | n |
| HadCM3 | Met Office Hadley Centre | UK | y | n | n |
| HadGEM2-AO | National Institute of Meteorological Research/Korea Meteorological Administration | Korea | y | y | y |
| HadGEM2-CC | Met Office Hadley Centre | UK | y | n | y |
| HadGEM2-ES | Met Office Hadley Centre, National Institute for Space Research (Brazil) | UK, Brazil | y | y | y |
| INM-CM4 | Institute for Numerical Mathematics | Russia | y | n | y |
| IPSL-CM5A-LR | Institut Pierre-Simon Laplace | France | y | y | y |
| IPSL-CM5A- MR | Institut Pierre-Simon Laplace | France | y | y | y |
| IPSL-CM5B-LR | Institut Pierre-Simon Laplace | France | y | n | y |
| MIROC-ESM | Japan Agency for Marine-Earth Science and Technology, Atmosphere and Ocean Research Institute (The University of Tokyo), and National Institute for Environmental Studies | Japan | y | y | y |
| MIROC-ESM- CHEM | Japan Agency for Marine-Earth Science and Technology, Atmosphere and Ocean Research Institute (The University of Tokyo), and National Institute for Environmental Studies | Japan | y | y | y |
| MIROC4h | Atmosphere and Ocean Research Institute (The University of Tokyo), National Institute for Environmental Studies, and Japan Agency for Marine-Earth Science and Technology | Japan | y | n | n |
| MIROC5 | Atmosphere and Ocean Research Institute (The University of Tokyo), National Institute for Environmental Studies, and Japan Agency for Marine-Earth Science and Technology | Japan | y | y | y |
| MPI-ESM-LR | Max Planck Institute for Meteorology | Germany | y | y | y |
| MPI-ESM-MR | Max Planck Institute for Meteorology | Germany | y | y | y |
| MPI-ESM-P | Max Planck Institute for Meteorology | Germany | y | n | n |
| MRI-CGCM3 | Meteorological Research Institute | Japan | y | y | y |
| MRI-ESM1 | Meteorological Research Institute | Japan | y | n | n |
| NorESM1-M | Norwegian Climate Centre | Norway | y | y | y |
| NorESMI-ME | Norwegian Climate Centre | Norway | y | y | y |

**Supplementary Table 2. List of instruments installed on the AWS**. Sensors installed on the glacier and on the rocky surface close to la Mortadelle.

| Variable[i], location | Type of Sensor | Height[ii], cm | Accuracy (s) according to the manufacturer |
|---|---|---|---|
| Air temperature, °C, Glacier | Vaisala HMP 45, aspirated[iii] | 200 | ±0.4°C |
| Relative humidity, %, Glacier | Vaisala HMP 45, aspirated[iii] | 200 | ±3 % |
| Wind speed, m $s^{-1}$, Glacier | Young 05103 | 210 | ±0.3 m $s^{-1}$ |
| Wind direction, deg, Glacier | Young 05103 | 210 | ±3 deg |
| Incident and reflected short-wave radiation, W $m^{-2}$, Glacier | Kipp&Zonen CMP3*, 0.305<□<2.8µm | 85 | ±10 %[iv] |
| Incoming long-wave radiation, W $m^{-2}$, la Mortadelle | Kipp&Zonen CG3*, 5<□<50 µm | 85 | ±10 %[iv] |
| Surface elevation, m, Glacier | Campbell acoustic gauge, SR50A | 160 | ±1 cm |
| Precipitation, mm (half-hourly), la Mortadelle | Précis mécanique 3029- | 85 | ±0.5 mm |

[i]Variables are recorded as half-hourly means over 10-s time-intervals except for wind direction which is an instantaneous value measured every 30 minutes.
[ii]Heights are variable but known with reasonable accuracy in summer thanks to manual measurements made every day, and sensor height is corrected assuming acoustic gauge measurements made during the rest of the year.
[iii]To prevent measurement errors due to radiation, Vaisala thermohygrometers are adequately shielded.
[iv]Expected accuracy for daily sums

## Supplementary Figures

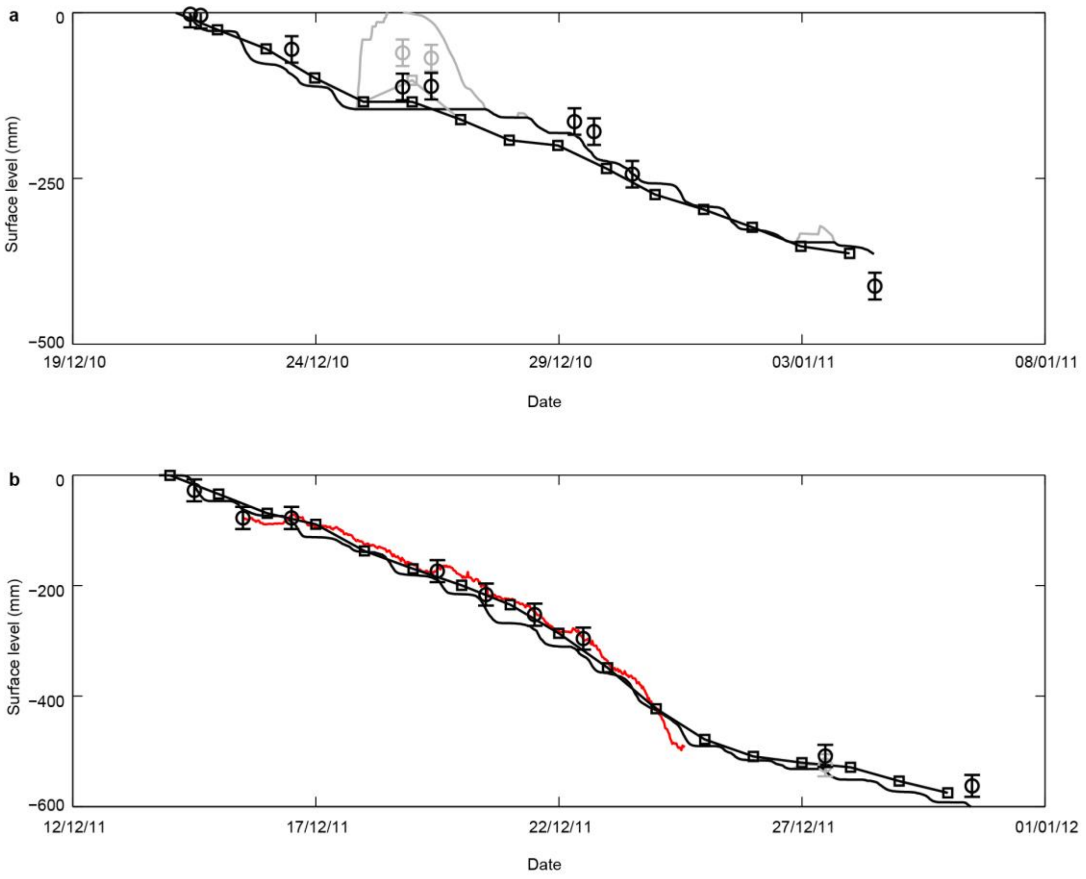


**Supplementary Figure 1. Validation of the PDD model at the AWS site.** Computed and measured surface height at the AWS during summer field campaigns in December 2010 (a) and December 2011 (b). Thick lines are the computed ice (in black) and snow (in gray) surface height using the full SEB approach. Lines with squares are the computed ice (in black) and snow (in gray) surface height using the PDD approach. Black and gray circles are the mean measured ice and snow surface height (respectively) at four stakes around the AWS. Red curve is the 30-minute surface elevation measured with an acoustic gauge at the AWS in 2011. Error bars are ±20 mm, which represent the mean standard deviation between stake measurements.

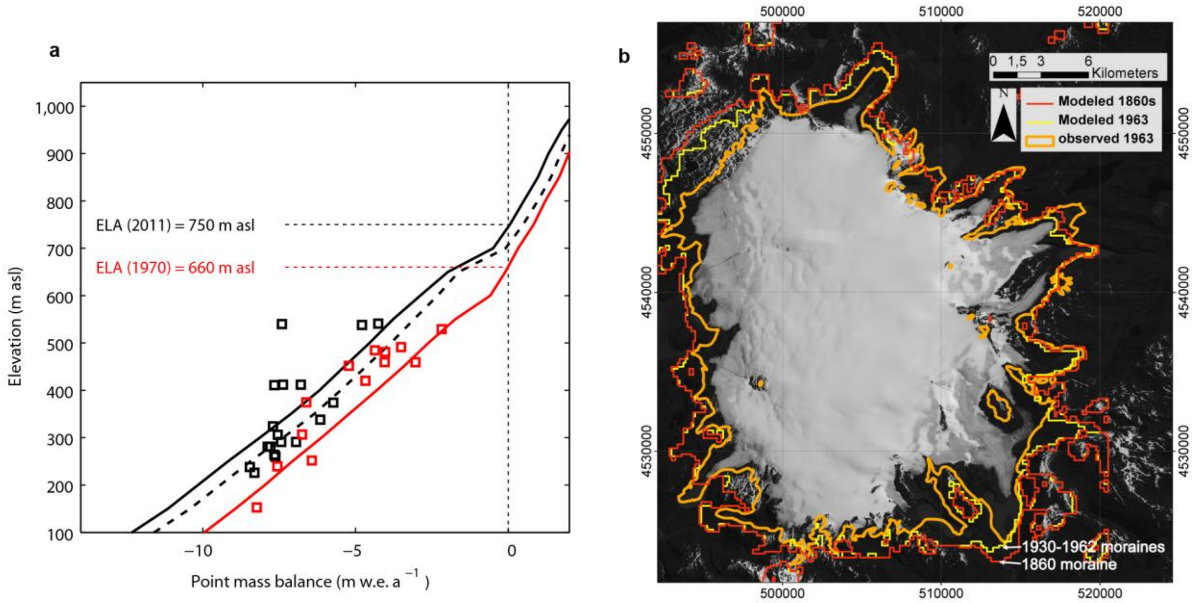


**Supplementary Figure 2**. **Distributed validation of the PDD model.** Mass balance validation. a) Measured (squares) and modelled (lines) surface mass balance of Ampère Glacier during the 1970-1974 period (red) and during the year 2011 (black). Continuous lines are the surface mass balance computed over 365 days, i.e. over March 15 to March 14 period. This period matches the timing of stake measurements in the 1970s. Discontinuous line is the modelled surface mass balance from January 1, 2011 to December 31, 2011, to compare with the stake measurements from December 31, 2011. b) Modelled glacier extents in 1930-62 (yellow) and in 1860 compared to the observations in 1963 (orange) and to observed Ampère glacier moraines from ref. 14. Background is the DEM computed from a stereoscopic pair of SPOT5 (ref 3) images taken in December 2009 used in this study (copyright CNES 2009, distribution Spot Image). Coordinate system is UTM42S. Map generated with ARCGIS 10.2 ArcInfo single use (http://www.esri.com/).

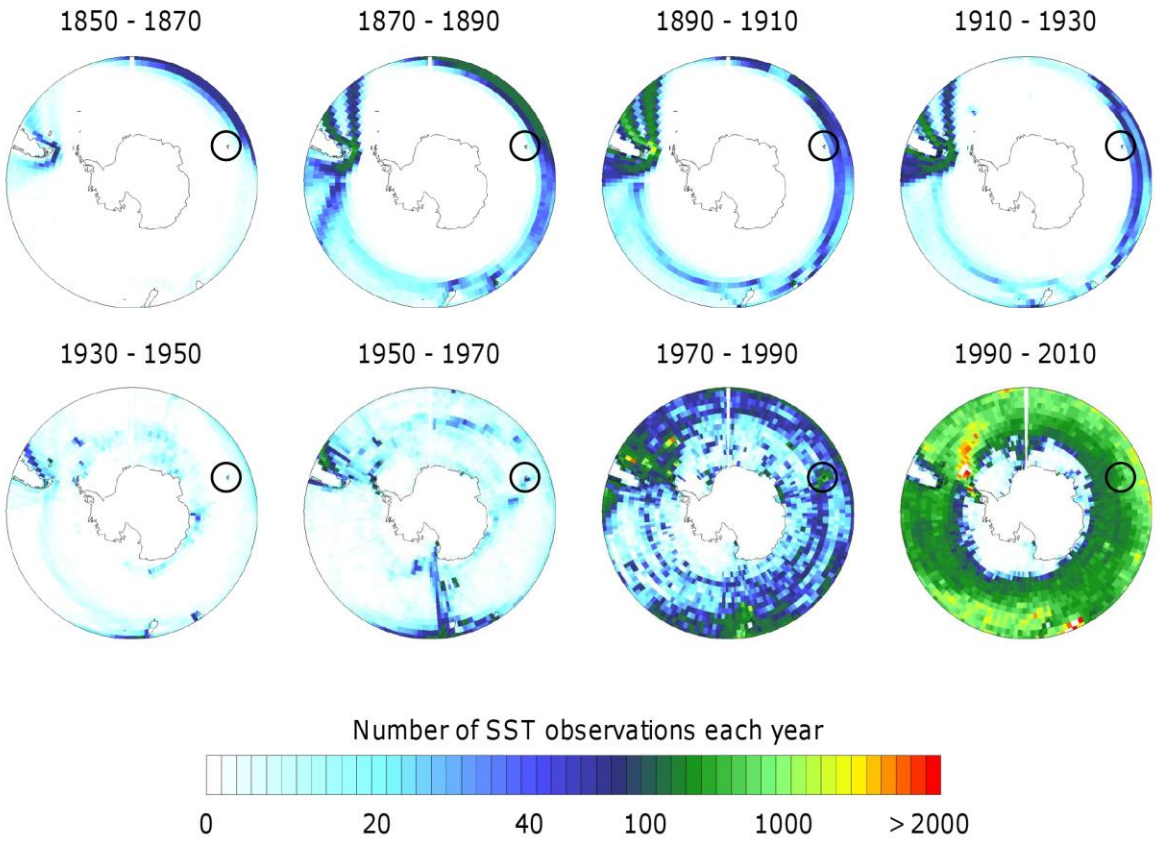


**Supplementary Figure 3. Number of SST observations since 1850.** Mean number of SST observations available each year in the Comprehensive Ocean-Atmosphere Data Set (COADS) dataset. Means are computed over 20-year intervals between 1850 and 2010. The Kerguelen Islands are located inside the black circle. Maps generated with Ferret v6.842 (http://www.ferret.noaa.gov/Ferret/).

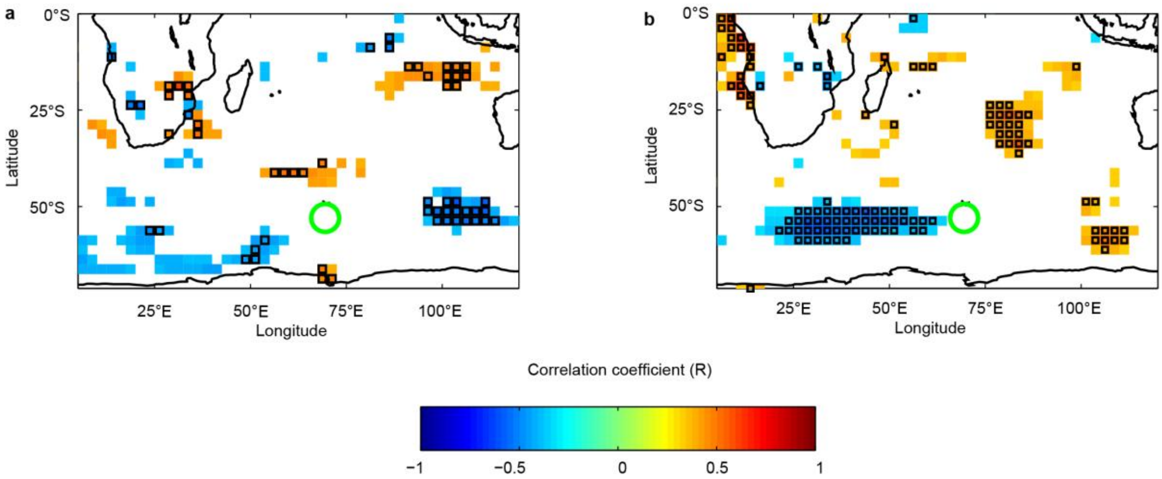


**Supplementary Figure 4. Present location of the storm track.** Correlation coefficient (*R*) between December and March (DJFM) ERA40 precipitation from the pixel located 350 km south from Kerguelen and ERA40 surface latent heat flux during DJFM. Surface latent heat flux is negative when evaporation occurs. Correlation maps are made before 1975 (a) and after 1975 (b). All time-series are detrended. Pixels where correlation is significant at 95% are in squared areas, at 90% are in colored areas. Pixels where correlation significance is lower than 90% are not shown. The point used for correlations is located inside the green circle. Maps generated with Matlab R2011b (www.mathworks.com/products/matlab/).

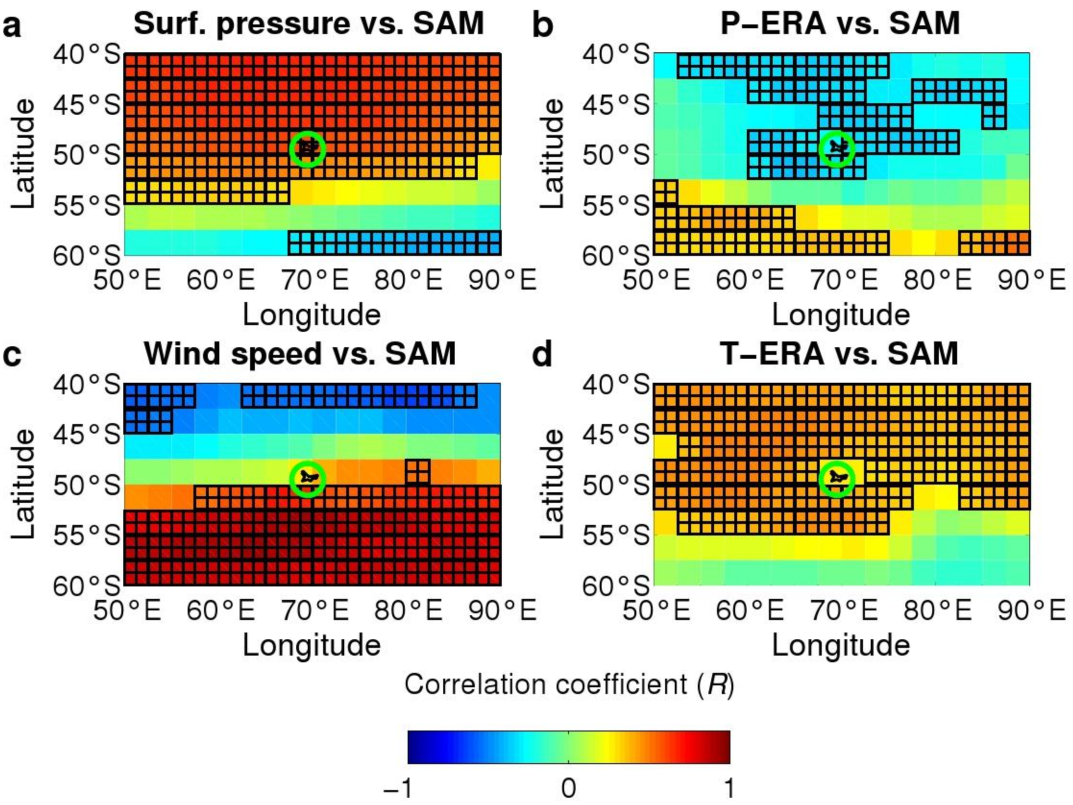


**Supplementary Figure 5. Impact of the SAM on the regional atmospheric features.** Correlation maps between the SAM-index anomalies and ERA-40's data on 1957-2002 period; a) surface pressure, b) precipitation, c) wind speed, and d) surface temperature. Pixels where correlation is significant at 95% are in squared areas. The Kerguelen Islands are located inside the green circle. Maps generated with Matlab R2011b (www.mathworks.com/products/matlab/).

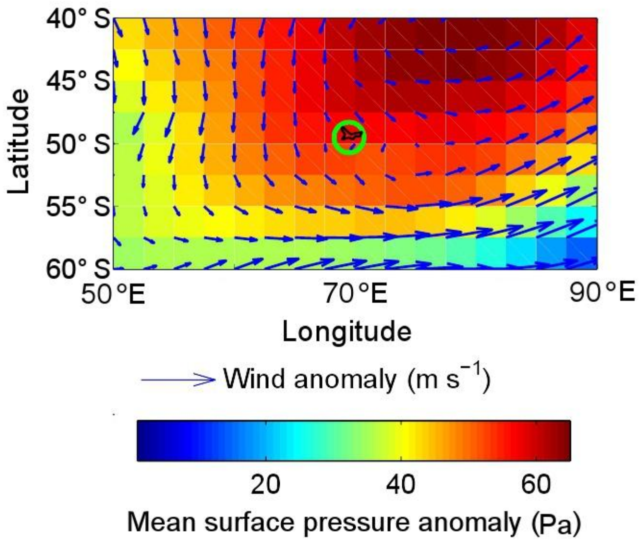


**Supplementary Figure 6. Anticyclone circulation anomaly around the Kerguelen Islands (circle).** Differences in pressure and wind speed (blue arrows) between 1960-1975 and 1975-1990 periods. The Kerguelen Islands are located inside the green circle. Map generated with Matlab R2011b (www.mathworks.com/products/matlab/).

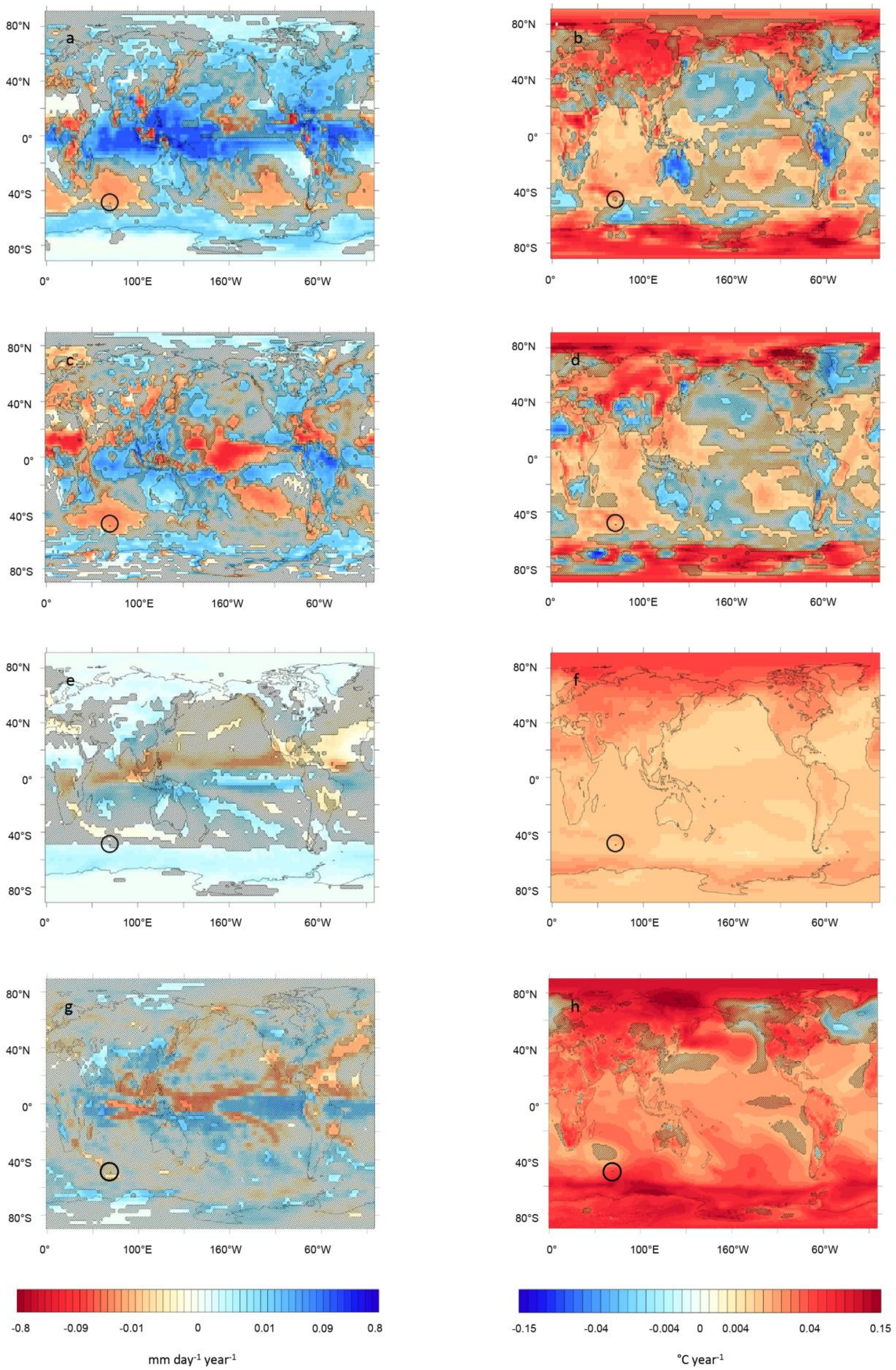

a
b
c
d
e
f
g
h
80°N
40°N
0°
40°S
80°S
0°
100°E
160°W
60°W
-0.8
-0.09
-0.01
0
0.01
0.09
0.8
mm day$^{-1}$ year$^{-1}$
-0.15
-0.04
-0.004
0
0.004
0.04
0.15
°C year$^{-1}$

**Supplementary Figure 7. Recent large scale precipitation (left) and temperature trends (right).** Precipitation and surface temperature trends for 1957-2002 in ERA-40 (a, b), NCEP1 (c, d) and CMIP5 (e, f) multi-model mean (MMM), and in the models showing (g) the highest drying (i.e. FIO-ESM model, with -4.34 x $10^{-3}$ mm $day^{-1}$ $a^{-1}$) and (h) the highest warming (CESM1-FASTCHEM, with +0.03341°C $a^{-1}$) at Kerguelen over 1950-2000. Shading indicates areas where the trend is not significant at the 95% confidence interval (Spearman test). Kerguelen's location is indicated by a black circle. Maps generated with Ferret v6.842 (http://www.ferret.noaa.gov/Ferret/).

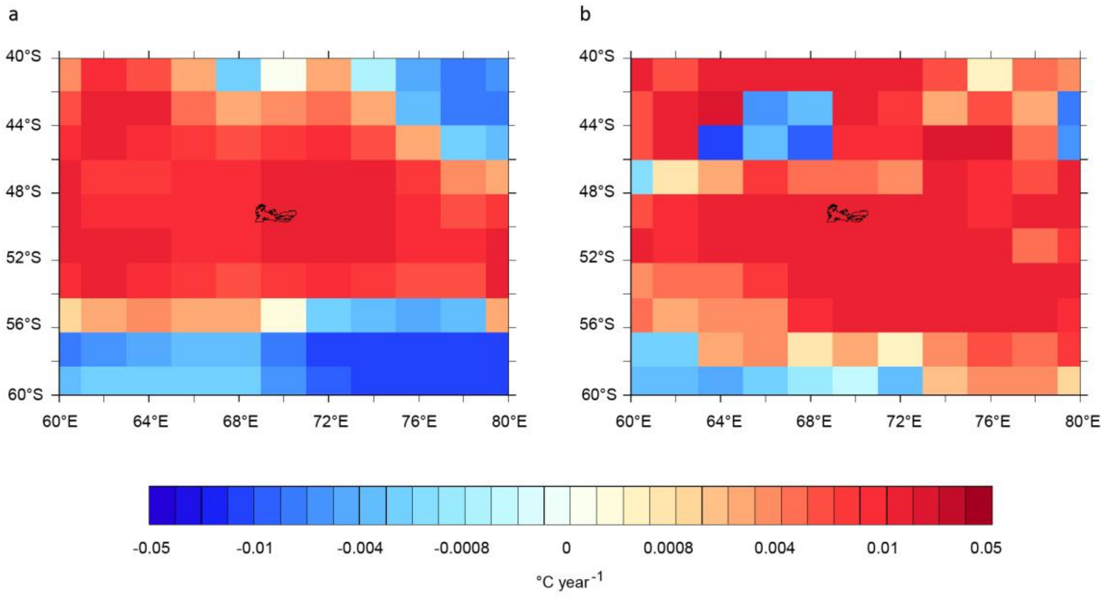


**Supplementary Figure 8**. **Sea Surface Temperature linear trends over 1982-2012**. a) HadSST2 data, b) AVHRR data. Each dataset has been re-gridded on the same 2°x2° regular grid. Map generated with Matlab R2011b (www.mathworks.com/products/matlab/).

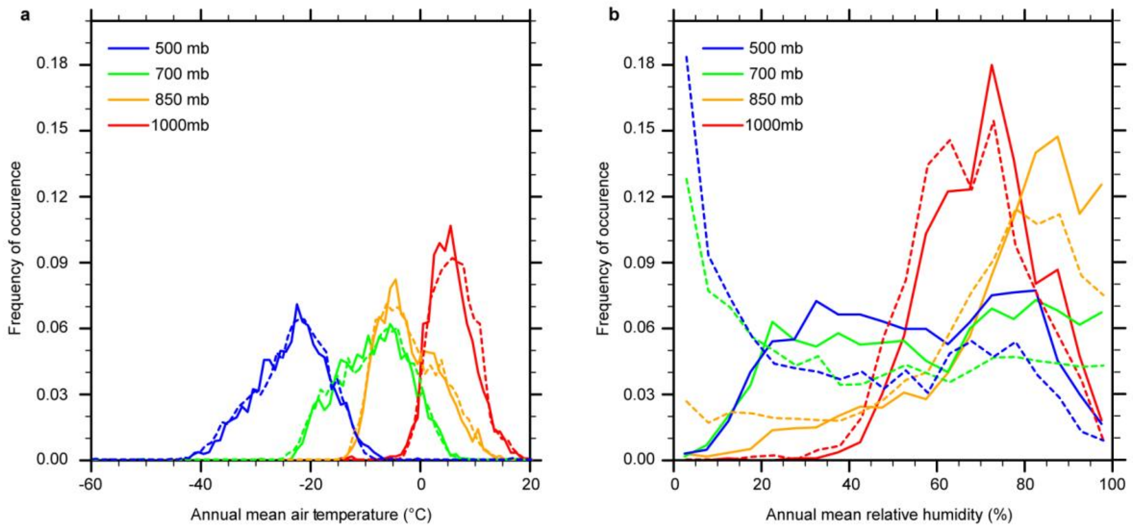


**Supplementary Figure 9. Temperature and humidity histograms from the Kerguelen Islands radiosonde (WMO ID 61998)**. Temperature (a) and relative humidity (b) histograms are presented for 1973-1982 (solid lines), and 2002-2011 (dashed lines). Data are represented at 500 mb (blue), 700mb (green), 850 mb (orange) and 1000 mb (red).

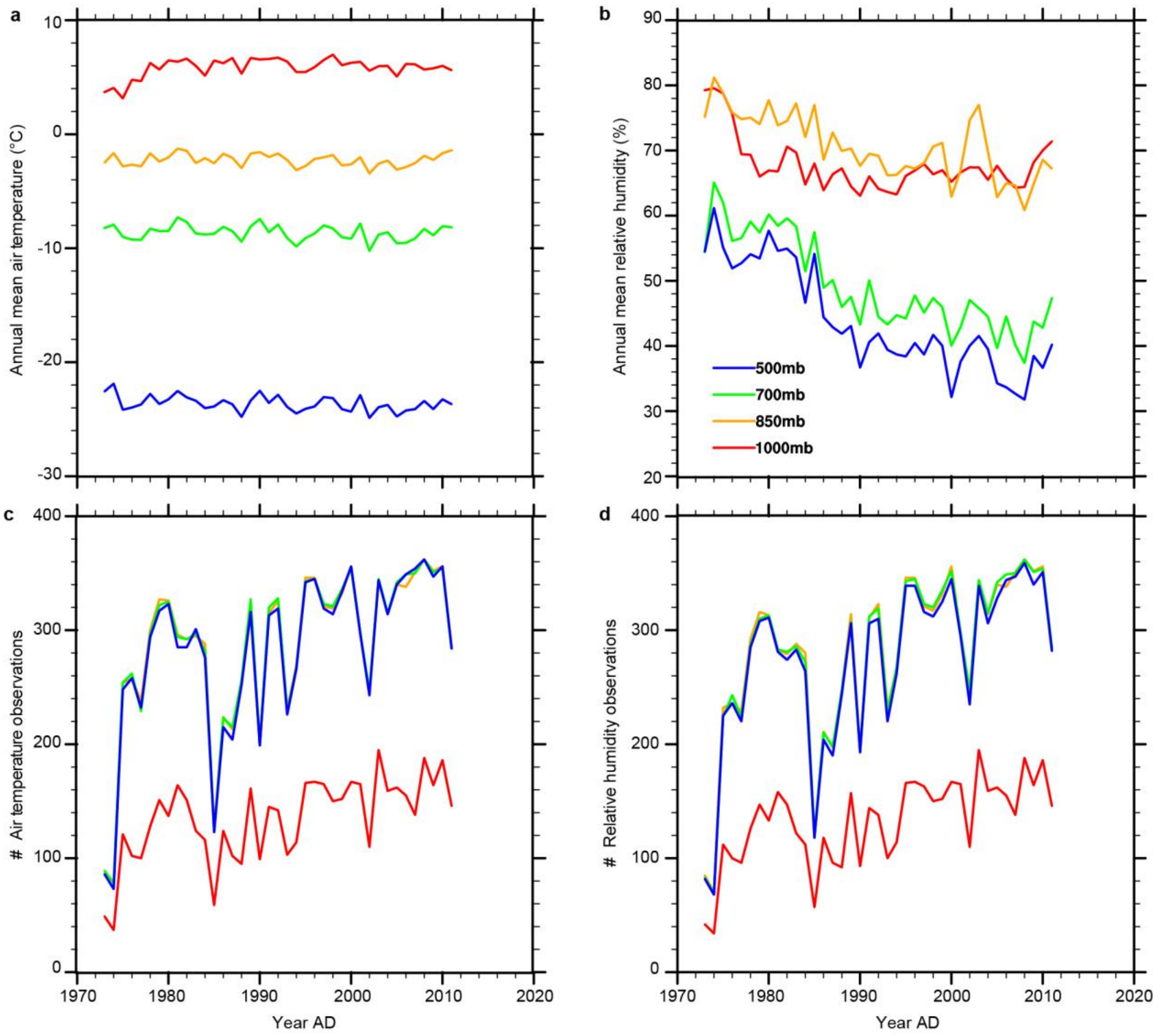


**Supplementary Figure 10. Temperature and humidity time-series from the Kerguelen Islands radiosonde (WMO ID 61998)**. Time-series are for a) annual mean air temperature at 500 mb (blue), 700 mb (green), 850 mb (orange) and 1000 mb (red), b) annual mean relative humidity at 500 mb, 700 mb, 850 mb and 1000 mb, c) number of air temperature observations per year at each elevation, d) number of relative humidity observations at each elevation.

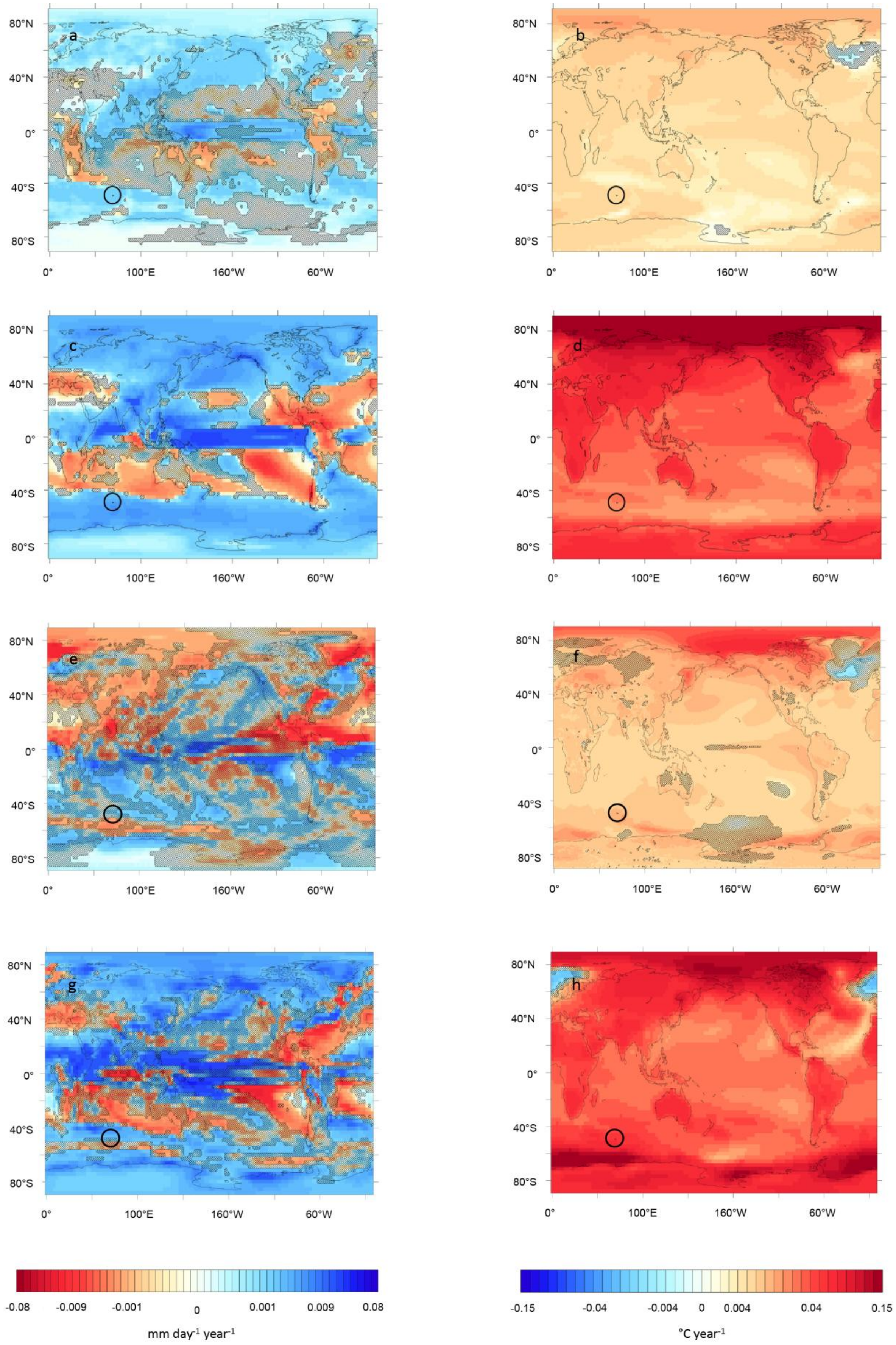
80°N
40°N
0°
40°S
80°S
0°
100°E
160°W
60°W
a
b
c
d
e
f
g
h
-0.08
-0.009
-0.001
0
0.001
0.009
0.08
mm day⁻¹ year⁻¹
-0.15
-0.04
-0.004
0
0.004
0.04
0.15
°C year⁻¹

**Supplementary Figure 11. Projected large scale precipitation (left) and temperature (right) trends.** Precipitation and surface temperature projected trends for 2006-2100 using the CMIP5 multi-model mean (MMM) for RCP 2.6 scenario (a, b) and RCP 8.5 scenario (c, d). (e, f, g, h) same as (a, b, c, d) but using the models showing the highest drying and warming over the Kerguelen Islands (e, f) for RCP 2.6 scenario (i.e. the FIO-ESM model (left), with -6.19 x $10^{-4}$ mm $day^{-1}$ $a^{-1}$; and the CESM1-CAM5 model (right), with +0.0176 °C $a^{-1}$) and (g, h) for RCP8.5 scenario (i.e. the BNU-ESM model (left), with +1.59 x $10^{-4}$ mm $day^{-1}$ $a^{-1}$; and the FIO-ESM model (right), with +0.0496 °C $a^{-1}$). Shading indicates areas where the trend is not significant at the 95% confidence interval (Spearman test). Kerguelen's location is indicated by a black circle. Please note the one-order magnitude difference between current (Supplementary Fig. 5) and projected (this Figure) precipitation trends scale. Maps generated with Ferret v6.842 (http://www.ferret.noaa.gov/Ferret/).

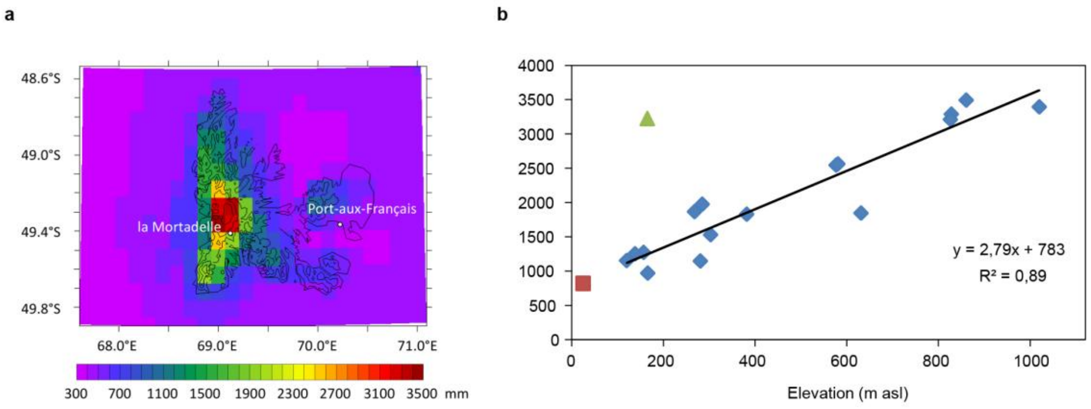


**Supplementary Figure 12**. **Modelled distribution of precipitation on CIC with the MAR model.** a) Map of 2011 modelled cumulative precipitation over the Kerguelen Islands with the MAR model. Black contours are terrain elevation (ETOPO1, represented with 300 m intervals) used to force the MAR model. Map generated with Ferret v6.842 (http://www.ferret.noaa.gov/Ferret/). b) Precipitation amounts from the MAR model as a function of elevation (blue points). Red square and green triangle are precipitation at PAF and at la Mortadelle respectively.

149
